# Longitudinal dynamics of gut microbiota in the pathogenesis of acute graft‐versus‐host disease

**DOI:** 10.1002/cam4.6557

**Published:** 2023-12-06

**Authors:** Ling Qi, Jie Peng, Xianbao Huang, Ting Zhou, Genmei Tan, Fei Li

**Affiliations:** ^1^ Center of Hematology The First Affiliated Hospital of Nanchang University Nanchang China; ^2^ Jiangxi Clinical Research Center for Hematologic Disease Nanchang China; ^3^ Institute of Lymphoma and Myeloma Nanchang University Nanchang China; ^4^ Clinical Medical College of Nanchang University Nanchang China

**Keywords:** cytokine, dynamics, graft‐versus‐host disease, gut microbiota

## Abstract

**Aim:**

The gut microbiota has been reported to be associated with acute graft‐versus‐host disease (aGvHD) in hematopoietic stem cell transplantation (HSCT). Dynamic surveillance of the microbiota is required to understand the detailed pathogenesis involved in the process of aGvHD.

**Methods:**

Fecal samples were collected prospectively at four timepoints, including pre‐HSCT (T1), graft infusion (T2), neutrophil engraftment (T3), and 30 days after transplantation (T4). Fecal samples were profiled by 16S ribosomal RNA gene sequencing to assess the microbiota composition.

**Results:**

From the T1 to T4 timepoint, the diversity of the gut microbiota decreased, and the dominant species also changed, with a decrease in the obligate anaerobic bacteria and a shift toward a “pathogenic community”. Compared with non‐aGvHD patients, aGvHD patients had a lower abundance of *Roseburia* at T1 and a higher abundance of *Acinetobacter johnsonii* at T2. Furthermore, *Acinetobacter johnsonii* was negatively correlated with the secretion of IL‐4 and TNF‐α. At T3, *Rothia mucilaginos* was demonstrated to be linked with a decreased risk of aGvHD, which was accompanied by decreased secretion of IL‐8. At T4, higher abundances of *Lactobacillus paracasei* and *Acinetobacter johnsonii* were identified to be related with aGvHD. *Lactobacillus paracasei* was associated with the downregulation of IL‐10, and *Acinetobacter johnsonii* was associated with the downregulation of IL‐2 and TNF‐α.

**Conclusions:**

Dynamic changes in gut microbiota composition and related cytokines were found to be related to aGvHD, including pathogenic or protective changes. These findings suggested that manipulation of gut microbiota at different timepoints might be a promising avenue for preventing or treating this common complication.

## 1 INTRODUCTION

Allogeneic hematopoietic stem cell transplantation (allo‐HSCT) remains a curative strategy for a variety of hematologic diseases. During the process, recipients are usually exposed to chemotherapy, broad‐spectrum antibiotics, and immunosuppressive agents, which are all known to affect the dynamic changes in the gut microbiota.^1^, ^2^, ^3^ There is growing evidence that the gut microbiota is linked to the prognosis of patients undergoing allo‐HSCT.^4^ Published studies on the gut microbiota in allo‐HSCT have indicated that the diversity of the gut microbiota is associated with the incidences of mortality and complications as well as the speed of immune reconstitution.^5^, ^6^, ^7^

Acute graft‐versus‐host disease (aGvHD) is a serious and even fatal complication after allo‐HSCT, and numerous studies have proven the predictive role of gut microbiota in aGvHD occurrence and related mortality.^8^, ^9^, ^10^, ^11^, ^12^, ^13^ The application of fecal microbiota transplantation in refractory aGvHD also supported the speculation that the gut microbiota might interact with the inflammatory storm during the pathological process of aGvHD,^14^, ^15^, ^16^ considering that the gut microbiota can influence normal innate and adaptive immunity.^7^ For the exploration of how gut microbiota triggers aGvHD, several researchers have similarly found that gut microbiota diversity, composition, and functional alterations were associated with the onset of aGvHD in both human and animal models.^17^, ^18^, ^19^, ^20^ However, the specific microbiota was inconsistent according to the above articles, probably due to the differences in races, transplant models, and timepoints of fecal collection among these studies.

Every timepoint during the process of allo‐HSCT has unique characteristics in terms of medication and immune alloreactivity, which have various effects on the diversity and composition of the gut microbiota. Most previous studies have evaluated gut microbiota at only one or a very limited number of timepoints during HSCT, but cross‐sectional studies cannot inform the directionality of the association between changes in gut microbiota and disease process. Thus, we comprehensively investigated the longitudinal dynamics of the gut microbiota at four key timepoints and the relationship with the occurrence of aGvHD through a prospective cohort.

## 2 METHODS

### 2.1 Study design

Adult patients (18 years or older) who received allo‐HSCT in our single center between 2020 and 2021 were enrolled in a prospective fecal collection protocol, which was approved by The First Affiliated Hospital of Nanchang University Ethics Committee. All study patients provided written informed consent for biospecimen collection and analysis. The study was conducted in accordance with the Declaration of Helsinki.

**FIGURE 1:**
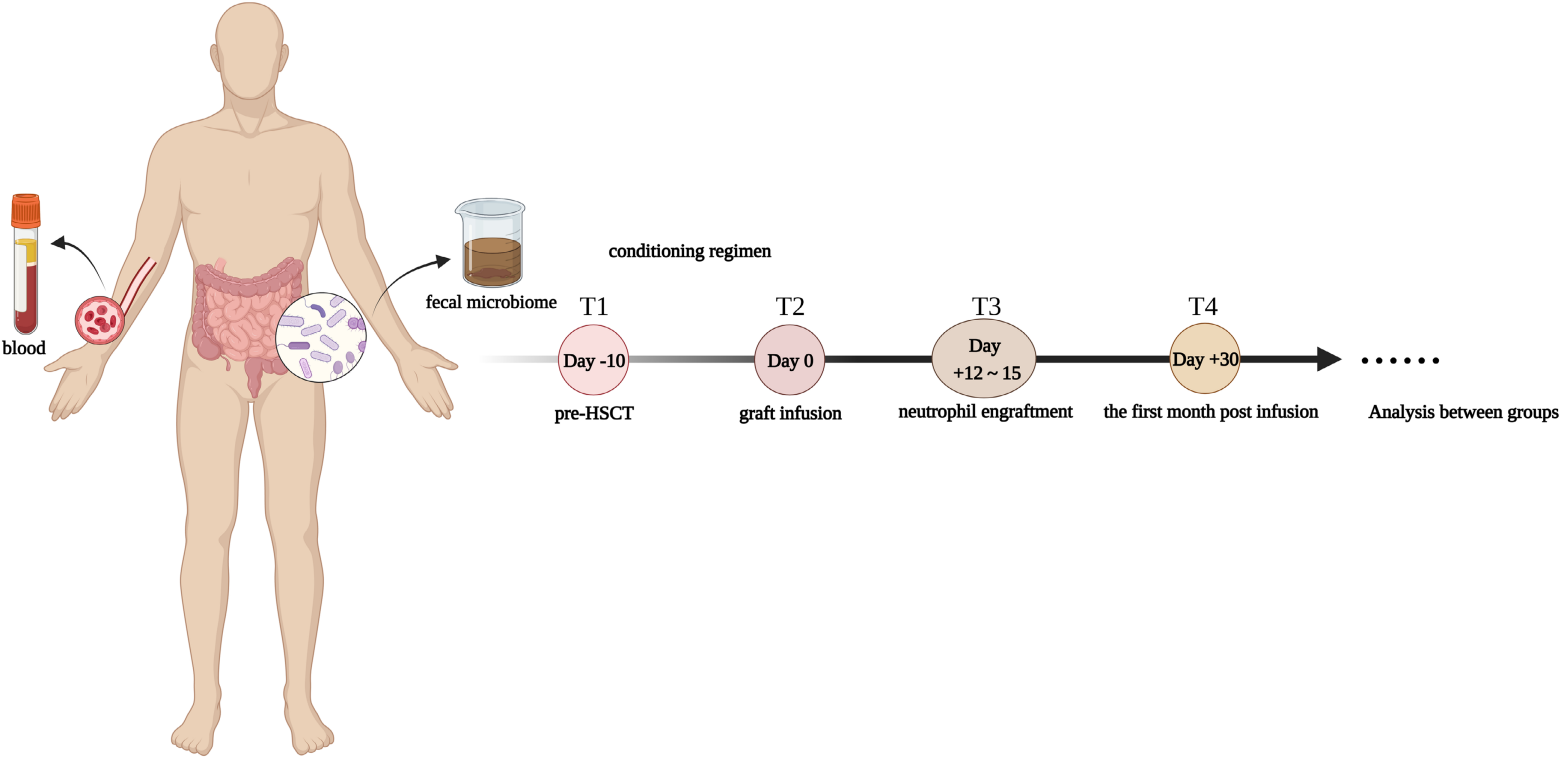
Flow chart of this experiment. We selected four timepoints to collect fecal and blood samples of patients, analyzed the dynamic changes of gut microbiota in different periods, and explored the correlation between gut microbiota and clinical predictors.

As indicated in Figure 1, fecal samples were collected prospectively at four timepoints. The T1 timepoint was before HSCT (baseline fecal samples, prior to HSCT conditioning regimens). The T2 timepoint was at the time of hematopoietic stem cell infusion (a pivotal timepoint after conditioning regimens and antibiotic gut decontamination). The T3 timepoint ranged from 12 to 15 days after graft infusion (systemic broad‐spectrum antibiotics are usually administered at this timepoint). The T4 timepoint was at 30 days after infusion (gut microbiota reacquire a configuration from Day 30^17^). All of these were excepted on days when patients had no feces within 3 days of these timepoints. Fecal samples were put into disposable sterile feces tubes and preserved at −80°C, shipped together with dry ice to the BGI Genomics Shenzhen, China, where microbiological analyses were performed. Blood samples were conducted concurrently from each individual timepoint for inflammation markers (C‐reactive protein, CRP, procalcitonin, PCT, erythrocyte sedimentation rate, and ESR), cytokines, and lymphocyte subset tests. We used ELISA to test the serum levels of the 12 selected cytokines, including IL‐1β, IL‐2, IL‐4, IL‐5, IL‐6, IL‐8, IL‐10, IL‐12p70, IL‐17, TNF‐α, α‐IFN, and γ‐IFN.

The patients were followed up to 100 days after transplantation and were divided into a non‐GvHD group and a GvHD group based on the Mount Sinai Acute GvHD International Consortium (MAGIC) criteria for acute GvHD.^21^

### 2.2 Sampling

#### 2.2.1 Genomic DNA extraction and library construction

The microbial community DNA was extracted using MagPure Stool DNA KF kit B (Magen) following the manufacturer's instructions. DNA was quantified with a Qubit fluorometer by using a Qubit® dsDNA BR Assay kit (Invitrogen), and the quality was checked by running an aliquot on a 1% agarose gel.

Variable regions V3–V4 of the bacterial 16S rRNA gene were amplified with degenerate PCR primers 341F (5’‐ACTCCTACGGGAGGCAGCAG‐3′) and 806R (5’‐GGACTACHVGGGTWTCTAAT‐3′). Both forward and reverse primers were tagged with Illumina adapter, pad, and linker sequences. PCR enrichment was performed in a 50 μL reaction containing 30 ng template, fusion PCR primer, and PCR master mix. PCR cycling conditions were as follows: 94°C for 3 min, 30 cycles of 94°C for 30 s, 56°C for 45 s, 72°C for 45 s and final extension for 10 min at 72°C for 10 min. The PCR products were purified with AmpureXP beads and eluted in elution buffer. Libraries were qualified by the Agilent 2100 bioanalyzer (Agilent). The validated libraries were used for sequencing on the Illumina MiSeq platform (BGI, Shenzhen, China) following the standard pipelines of Illumina and generating 2 × 300 bp paired‐end reads.

### 2.3 GvHD prophylaxis and antibiotic routine

Intensive GvHD prophylaxis consisted of cyclosporine A (CsA), methotrexate (MTX), mycophenolate mofetil (MMF), and anti‐thymocyte globulin (ATG). Antibiotic gut decontamination during the conditioning regimen was routinely performed with oral levofloxacin. Antiviral and antifungal prophylaxis was administered with ganciclovir/acyclovir and posaconazole/caspofungin from the beginning of the conditioning regimen. Sulfamethoxazole and trimethoprim were administered prophylactically for Pneumocystis carinii pneumonia. In cases of fever or clinical signs of infections, piperacillin/tazobactam, cefoperazone/sulbactam, imipenem/cilastatin, vancomycin, or other antibiotics were commenced according to culture‐based results or clinical presentation.

### 2.4 Data analyses

Raw reads were filtered to remove adaptors and low‐quality and ambiguous bases, and then paired‐end reads were added to tags by the Fast Length Adjustment of Short reads program (FLASH, v1.2.11)^22^ to obtain the tags. The tags were clustered into OTUs with a cutoff value of 97% using UPARSE software (v7.0.1090),^23^ and chimera sequences were compared with the Gold database using UCHIME (v4.2.40)^24^ for detection. Then, OTU representative sequences were taxonomically classified using Ribosomal Database Project (RDP) Classifier v.2.2 with a minimum confidence threshold of 0.6 and trained on the Greengenes database v201305 by QIIME v1.8.0.^25^ USEARCH_global^26^ was used to compare all tags back to OTUs to obtain the OTU abundance statistics table of each sample. Alpha and beta diversity were estimated by MOTHUR (v1.31.2)^27^ and QIIME (v1.8.0) at the OTU level, respectively. Sample clustering was conducted by QIIME (v1.8.0) based on UPGMA. Barplot and heatmap of different classification levels were plotted with R package v3.4.1 and R package “gplots”, respectively. In addition, LEfSe cluster or LDA analysis was conducted by LEfSe. Significant species were determined by R (v3.4.1) based on the Wilcox test or Kruskal test. Principal component analysis (PCA) and nonmetric multidimensional scaling (NMDS) dimensionality reduction analysis were performed using the R packages “abe4” and “vegan”. A PCoA (principal coordinates analysis) plot was plotted with QIIME (v1.80). Spearman's test was used for correlation analysis.

### 2.5 Statistical analysis

SPSS software 22.0 (IBM Corporation) and R version 4.2.2 software (The R Foundation for Statistical Computing) were used for statistical analysis. Overall survival (OS) was calculated from the date of transplantation to death due to any causes. The chi‐squared test, *t*‐test, or Mann–Whitney *U*‐test were used for descriptive statistical analyses. The Kaplan–Meier method was used to estimate OS, and differences among curves were evaluated by the log‐rank test. Cumulative incidences of malignancy relapse, transplant related mortality (TRM), and GvHD were calculated by accounting for competing risks using the Gray model. Parameters which were potentially significant or those with *p* < 0.2 by univariate analysis were entered into a multivariate analysis using a Cox proportional hazards model. *p* < 0.05 was considered statistically significant.

## 3 RESULTS

### 3.1 Patient characteristics

The patients enrolled in this study were required to have at least three samples for evaluation, and a total of 226 stool samples from 61 adult patients were analyzed eventually. The characteristics of the total cohort, GvHD and non‐GvHD groups are summarized in Table 1. There were 35 male and 26 female patients, and the median age (range) was 32 (18–60) years. Patients with either malignant (48/61) or nonmalignant (13/61) diseases were included. Regarding the disease status of hematological malignancy at transplant, 42 (68.85%) patients were in complete remission (CR), and 6 (9.84%) patients were non‐CR. A total of 25 (40.98%) patients underwent human leucocyte antigen‐matched sibling donor transplantation, and 36 (59.02%) patients were from haploidentical donors. All patients were treated with a myeloablative conditioning regimen and non‐T‐cell depletion in vitro strategy. Concerning the sources of stem cells, 42 (68.85%) patients received peripheral blood stem cells (PBSCs) plus bone marrow cells, while 19 (31.15%) patients received PBSCs. Multivariate analysis showed that baseline factors had no influence on the incidence of acute GvHD (Table S1).

**TABLE 1:** The clinical characteristics of 61 patients.

Parameters	Total (*n* = 61)	GvHD (*n* = 34)	Non‐GvHD (*n* = 27)	*p‐*Value
Sex				
Male	35	21 (61.8%)	14 (51.9%)	0.437
Female	26	13 (38.2%)	13 (48.1%)	
Median age, years (range)	32 (18–60)	32.5 (18–57)	31 (18–60)	0.441
Primary disease				
AML	26	16 (47.1%)	10 (37.0%)	0.105
ALL	17	11 (32.4%)	6 (22.2%)	
MPAL	1	0	1 (3.7%)	
MDS	3	3 (8.8%)	0	
Lymphoma	1	0	1 (3.7%)	
SAA	13	4 (11.8%)	9 (33.3%)	
Stem cell source				
BM + PB	42	24 (70.6%)	18 (66.7%)	0.743
PB	19	10 (29.4%)	9 (33.3%)	
Donor				
Matched sibling	25	12 (35.3%)	13 (48.1%)	0.311
Haploidentical	36	22 (64.7%)	14 (51.9%)	
Conditioning				
Bu/Cy/ATG ± Mel	39	23 (67.6%)	16 (59.3%)	0.306
Bu/Cy ± Mel	15	9 (26.5%)	6 (22.2%)	
Cy/ATG	7	2 (5.9%)	5 (18.5%)	
GVHD prophylaxis				
CsA + MMF + MTX + ATG	46	25 (73.5%)	21 (77.8%)	0.702
CsA + MMF + MTX	15	9 (26.5%)	6 (22.2%)	
Donor‐recipient gender				
Female to male	12	8 (23.5%)	4 (14.8%)	0.395
Others	49	26 (76.5%)	23 (85.2%)	
Disease status at transplant (hematological malignancy)				
CR	42	26 (76.5%)	16 (59.3%)	0.121
Non‐CR	6	4 (11.8%)	2 (7.4%)	

Abbreviations: ALL, acute lymphoblastic leukemia; AML, acute myeloid leukemia; ATG, anti‐thymocyte globulin; CR, complete remission; CsA, cyclosporine A; GvHD, graft versus host disease; MDS, myelodysplastic syndrome; MMF, mycophenolate mofetil; MPAL, mixed phenotype acute leukemia; MTX, methotrexate; SAA, severe aplastic anemia.

### 3.2 Changes in gut microbiota diversity in allo‐HSCT

**FIGURE 2:**
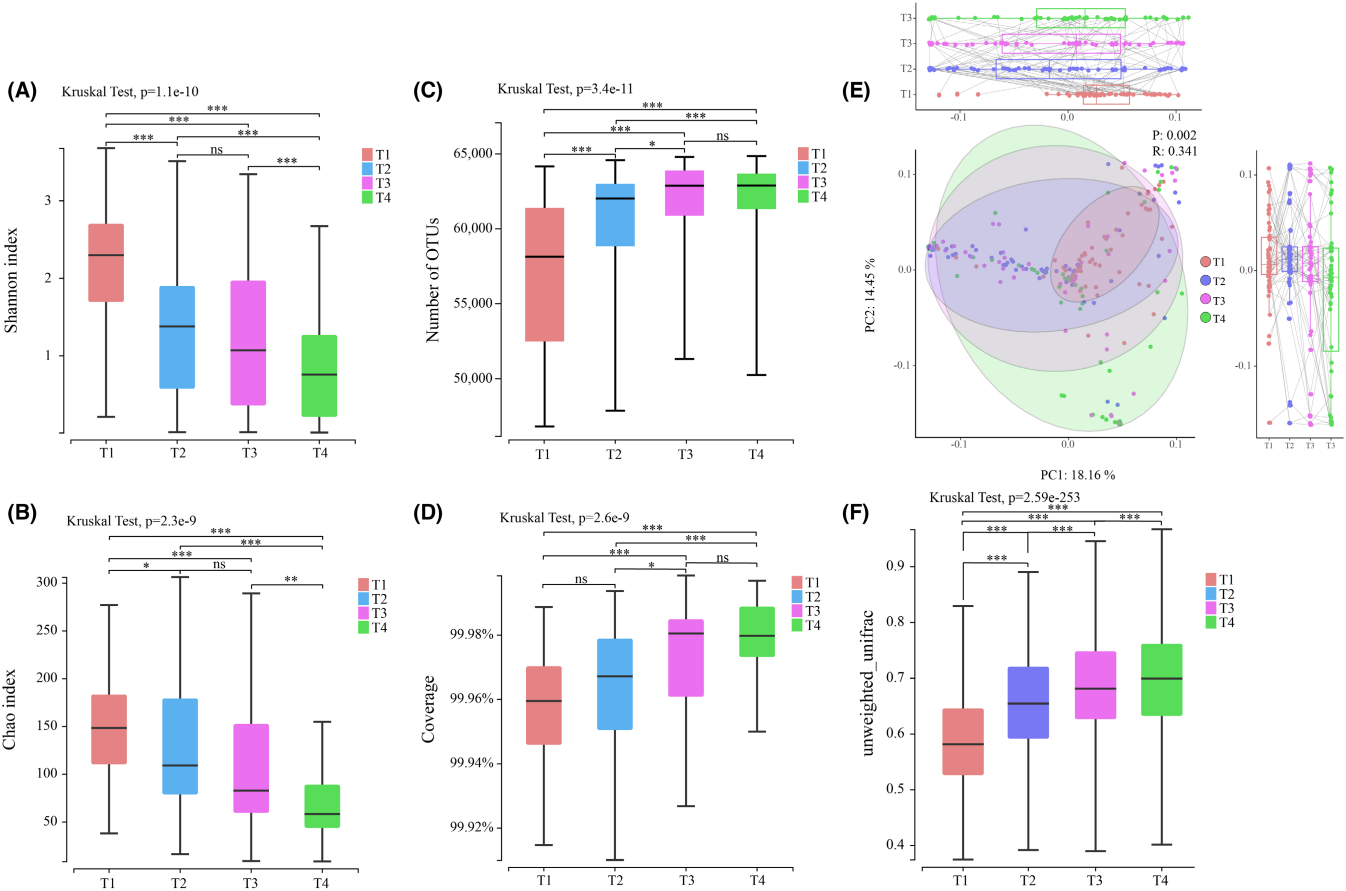
Changes of gut microbiota diversity during allo‐HSCT. Alpha diversity indexs of gut microbiota: (A) Shannon index, (B) Chao index, (C) Numbers of OTUs, (D) Coverage. Beta diversity indexs of gut microbiota: (E) PCA dimension reduction analysis, (F) unweighted UniFrac based on phylogenetic trees. (*p < 0.05, **p < 0.01, ***p < 0.001, ns means no significant difference).

The dynamic changes in gut microbiota diversity in allo‐HSCT during the T1–T4 period were assessed. Alpha diversity was used to reflect the changes in community diversity (Shannon index) and community richness (Chao index) (Figure 2A,B). From T1 to T4, the Shannon index and Chao index gradually decreased (*p* < 0.05), indicating that the community diversity and community richness decreased, which means that the number of microbial species in the gut gradually decreased. In addition, the numbers of OTUs gradually increased (Figure 2C). The coverage index was stable between 0.9996 and 0.9998, with mild difference and high authenticity of the results (Figure 2D).

Since alpha diversity focused on a single individual and did not involve comparisons between individuals, we used beta diversity to further characterize microbial composition differences between different periods. PCA was used for dimensionality reduction analysis (Figure 2E). The results indicate that there are significant differences between T1 and T2 and T3 on the PC1 dimension and significant differences between T4 and T1 and T2 on the PC2 dimension. Overall, from T1 to T4, the degree of difference within the group gradually increased, and the distribution changed from concentrated to scattered, which means that the dominant species changed in the process of allo‐HSCT. In addition, to analyze beta diversity among four timepoints, we plotted boxplots using unweighted UniFrac based on phylogenetic trees (Figure 2F). We can conclude that beta diversity increased gradually from T1 to T4 (*p* < 0.05), which indicates that the gut microflora of patients became increasingly different, which is in accordance with the results obtained above. Next, we wanted to further explore what was exactly changed in the composition of the gut microbiota.

### 3.3 Changes in gut microbiota composition in allo‐HSCT

**FIGURE 3:**
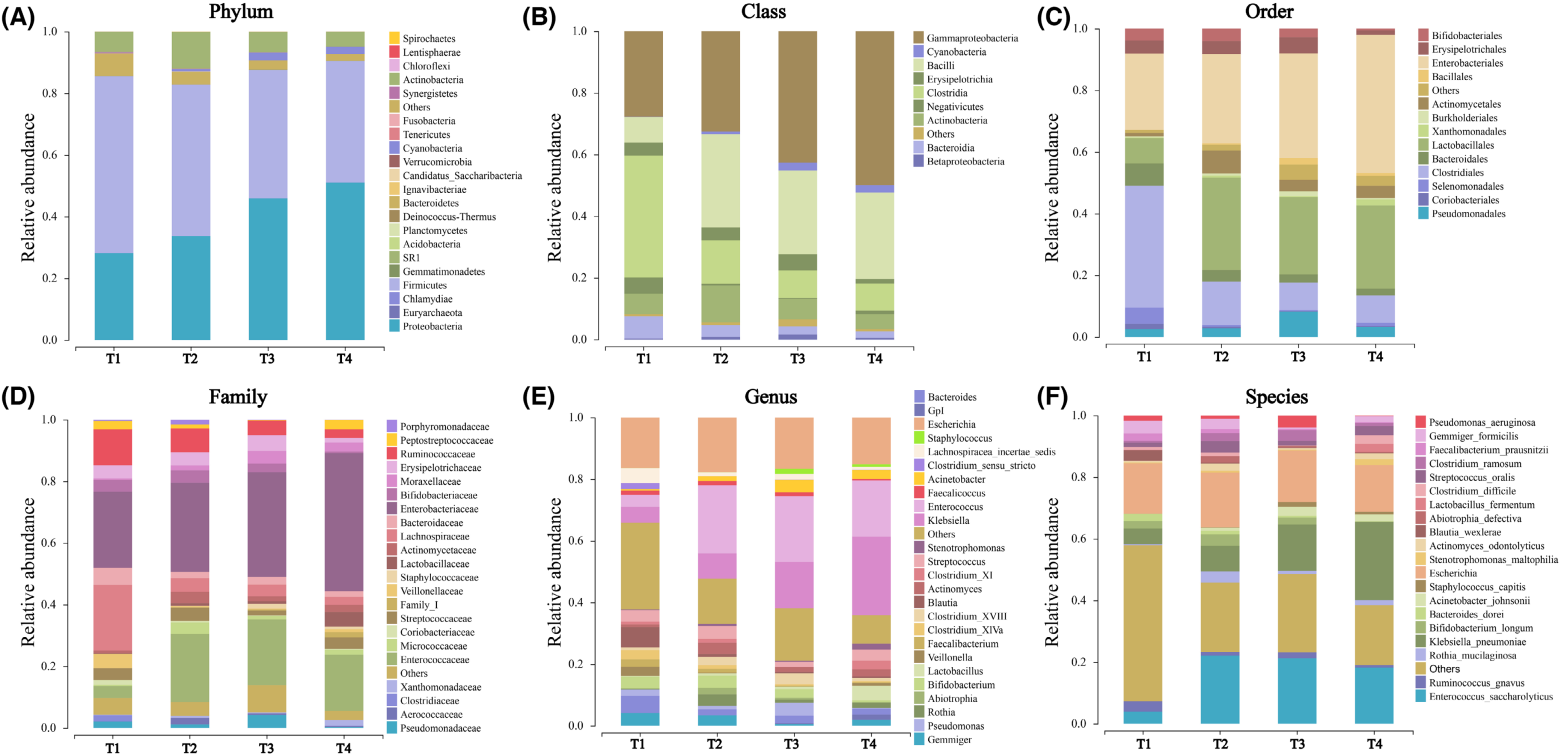
Changes in gut microbiota composition during allo‐HSCT. The level of (A) Phylum, (B) Class, (C) Order, (D) Family, (E) Genus and (F) Species.

**FIGURE 4:**
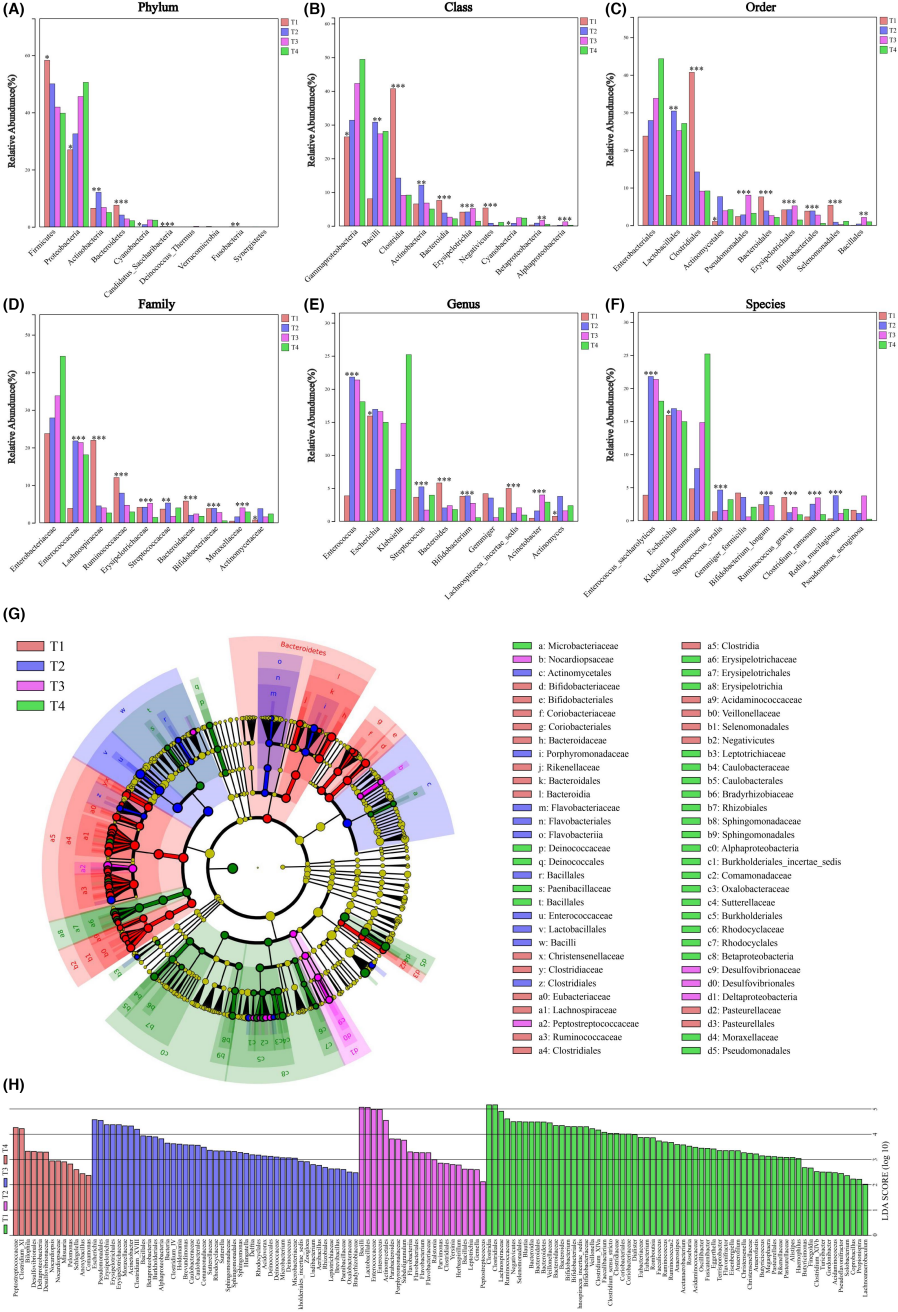
Changes in dominant gut microflorae during allo‐HSCT. In the level of (A) Phylum, (B) Class, (C) Order, (D) Family, (E) Genus, (F) Species, the top 10 gut microflorae were analyzed according to different stages. (G–H) Cladogram diagram was used to show the phylogenetic distribution of gut microflorae in different stages of allo‐HSCT. (G) Different colored were as represent different stages of allo‐HSCT. Circles represented the phylogenetic level from phylum to genus. The diameter of each circle is proportional to the richness of corresponding microflorae. (H) Indicative species with LDA≥2 in different periods. LDA: Latent Dirichlet Allocation.

A total of 1482 OTUs were annotated for follow‐up analysis, including gut microbiota from 21 phyla, 42 classes, 66 orders, 148 families, 405 genera, and 590 species. During the process of HSCT, at the phylum level, the OTU proportion of Firmicutes decreased, while that of Proteobacteria increased. At the class level, the OTU proportion of Clostridia decreased, while that of Gammaproteobacteria increased. At the order level, the OTU proportion of Clostridiales decreased while that of Enterobacteriales increased. At the family level, the OTU proportions of Lachnospiraceae and Ruminococcaceae decreased, while that of Enterobacteriaceae increased. At the genus level, the OTU proportion of Klebsiella increased, and at the species level, the OTU proportion of Klebsiella pneumoniae increased. The OTU proportion reduction in the phylum Firmicutes was mainly due to the decrease in the families Lachnospiraceae and Ruminococcaceae, while the OTU proportion increase in the phylum Proteobacteria was mainly due to the increase in the species Klebsiella pneumoniae (Figure 3). Meanwhile, the top 10 gut microbiota at the phylum, class, order, family, genus, and species levels were analyzed according to different timepoints, and the results are shown in Figure 4A–F.

In particular, from T1 to T2, the OTU proportion of the order Lactobacillales and family Enterococcaceae increased significantly and then remained relatively stable from T2 to T4. Meanwhile, the OTU proportion of the family Lachnospiraceae and class Clostridia showed a sharp decline from T1 to T2. At the same time, we focused on some pathogenic bacteria and complication‐related microbiota. We found that the OTU proportion of Escherichia remained relatively stable, and Acinetobacter increased continuously from T1 to T3 and then decreased from T3 to T4. From T1 to T2, the OTU proportion of *Stenotrophomonas maltophilia* showed an upward trend, and from T2 to T4, it showed a downward trend.

To further explore the species with significant differences in richness between groups (biomarkers), we applied LEfSe (linear discriminant analysis) to explore the biomarkers (LDA >2) from the phylum to genus level and displayed them with a cladogram diagram (Figure 4G,H). In Figure 4G, from the inside to the outside, the phylum, class, order, family, genus, and species are listed in order. Each node represents a microflora, and the corresponding color indicates that the abundance of the microflora had significant differences in the corresponding period. In terms of the number of biomarkers, T1 (63) had the most species with significant differences in richness, followed by T3 (44), T2 (21), and T4 (13) (Figure 4H). From T1 to T4, the proportion of biomarkers of the phylum Firmicutes gradually decreased from 71% to 23%, while that of the phylum Proteobacteria gradually increased from 5% to 62%. Specifically, the most significant biomarker enriched in the T1 period was Clostridiales, while the most significant biomarkers at T2, T3, and T4 were Bacilli, Escherichia, and Peptostreptococcaceae, respectively.

### 3.4 The microbiota associated with aGvHD at different timepoints of allo‐HSCT

The cumulative incidence (CI) of Grade II–IV aGvHD were 36.1 ± 0.4%, 49.2 ± 0.4% and 55.7 ± 0.4% on Day 30, 60, and 100 posttransplantation (Figure S1A). The CI of Grade III–IV aGvHD were 19.7 ± 0.3%, 29.5 ± 0.3%, and 36.1 ± 0.4% on Day 30, 60 and 100 posttransplantation (Figure S1B). Besides, the details about GvHD parameters have also been described in Table S2.

**FIGURE 5:**
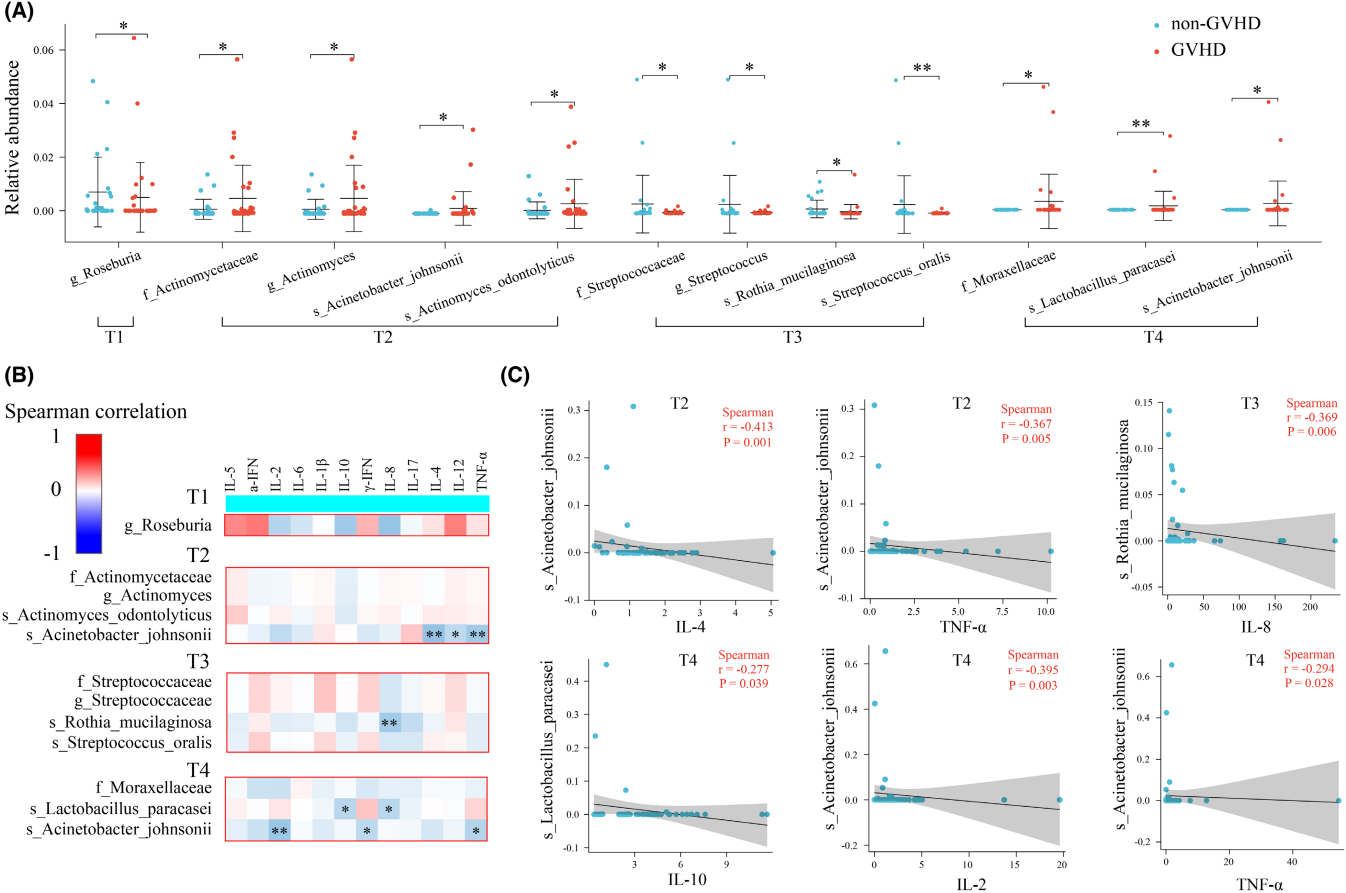
The relationship between gut microbiota and aGVHD. (A) Differential microflorae in different aGVHD groups at four timepoints. (B) Heatmap of the correlation between differential microbiota and cytokines. (C) The correlation curve of differential microflorae with cytokines. (*p < 0.05, **p < 0.01, ***p < 0.001 ns means no significant difference).

How the gut microbiota is involved in the pathologic process of aGvHD onset is not yet fully known. First, we analyzed the correlation between gut microbiota and immune cell subsets, cytokines and other clinical predictors at four timepoints (Figure S2). Immune cells were not measured at T1 because transplantation had not yet begun and the immune cells were patient‐derived. The immune cells were not detectable at T2 timepoint due to myeloablative chemotherapy. Second, to further explore the relationship between gut microbiota and aGvHD, we compared the relative abundance of gut microbiota in aGvHD patients and non‐aGvHD patients at the family, genus, and species levels in each period. Gut microbiota with an average relative abundance greater than 0.5% were included in further analysis. The results showed that, compared with non‐aGvHD patients, aGvHD patients have lower abundance of Roseburia in T1 period, higher abundance of Actinomycetaceae, Actinomyces, Acinetobacter_johnsonii, Actinomyces_odontolyticus in T2 period, lower abundance of Streptococcaceae, Streptococcus, *Rothia mucilaginosa*, *Streptococcus oralis* in T3 period, and a higher abundance of Moraxellaceae, *Lactobacillus paracasei*, *Acinetobacter johnsonii* in T4 period. (Figure 5A).

Furthermore, we analyzed the correlation between these differential microbiota and cytokines (Figure 5B). We found that in the T2 period, Acinetobacter johnsonii was negatively correlated with IL‐4 and TNF‐α. In the T3 period, *Rothia_mucilaginosa* was negatively correlated with IL‐8. In the T4 period, *Lactobacillus paracase*i was negatively correlated with IL‐10, and *Acinetobacter johnsonii* was negatively correlated with IL‐2 and TNF‐α. The details are shown in Figure 5B,C.

When analyzing the differences in the overall levels of cytokines between the GvHD and non‐GvHD groups, we found that IL‐8 at the T2 timepoint, IL‐17, IFN‐α, IL‐2, IL‐1, IL‐8, TNF‐α at the T3 timepoint were obviously elevated, and IL‐10 at the T4 timepoint was decreased with significance in GvHD group.

In addition, the microbiota associated with aGvHD at different timepoints did not affect other transplant outcomes, including OS, relapse and TRM (Table S3).

## 4 DISCUSSION

Through a prospective cohort, we demonstrated the longitudinal dynamics of gut microbiota during the process of allo‐HSCT and analyzed the GvHD‐related microbiota as well as cytokines at four different timepoints, including preconditioning (T1), the day of graft infusion (T2), the period of engraftment (T3), and Day 30 posttransplantation (T4).

Our results revealed that the diversity of the gut microbiota decreased within 1 month after transplantation, and the lowest alpha diversity was observed on Day 30 after infusion. Consistent with published studies,^28^, ^29^, ^30^ the gut microbiota among recipients experienced radical changes during different periods of allo‐HSCT due to the injury of chemotherapy and antibiotics and the tissue damage of immune alloreactivity. Previous observations have also shown that the community richness and diversity decreased, and the dominant species also changed; thus, certain species dominating post‐HSCT may be involved in promoting aGvHD.^4^, ^9^, ^11^, ^31^, ^32^, ^33^ The relationship between microbiota patterns and GvHD occurrence has been explored in numerous studies.^8^, ^9^, ^10^, ^11^, ^12^, ^13^, ^17^, ^18^, ^19^, ^20^ Stein‐Thoeringer et al. provided evidence that *Enterococcus* expansion promoted the pathogenesis of GvHD in both mice and allo‐HCT patients.^9^ Payen et al. proved that gut microbiota alterations were linked to the severity of GvHD, and *butyrate* represented a potential diagnostic marker of GvHD at all timepoints.^19^ In our study, the microbiota diversity did not differ at each timepoint between the GvHD and non‐GvHD groups, but the reduced microbiota diversity deprived the phylum Firmicutes (such as Lachnospiraceae, Ruminococcaceae and Clostridia) with a shift toward a “pathogenic community” (includingLactobacillales, Enterococcaceae, Acinetobacter, and *Stenotrophomonas maltophilia*). As previously reported, this so‐called anti‐inflammatory Clostridia might exert a counteracting effect on aGvHD onset and progression, upregulating Treg cells through the production of the SCFA butyrate.^17^, ^18^, ^34^ Meanwhile, aGvHD has also been attributed to major fluctuations in gut microbiota, which featured a general loss of diversity and expansion of Enterobacteriales (including *Escherichia*, *Klebsiella*, and *Enterobacter*), Lactobacillales (including *Lactobacillus*, *Enterococcus*, *and Streptococcus*), Proteobacteria and Akkermansia.^35^

When the analysis was conducted based on the different timepoints, it was important to explore whether the changes in the gut microbiota preceded or followed the development of complications or both. Thus, useful in determining whether gut microbiota were drivers or passengers. Consequently, we focused on the changes at each timepoint. We noted abundance differences between the two groups at T1. As all patients had received antibiotics during the 3 months before conditioning, the difference at T1 may be due to the degree of infection and the use of antibiotics preconditioning. We noticed that Lactobacillales and Enterococcaceae increased significantly from T1 to T2 and then remained relatively stable from T2 to T4. In addition, Lachnospiraceae and Clostridia showed a sharp decline from T1 to T2, while the decline slowed from T2 to T4. According to the order of LDA, the most significant biomarker enriched in T1 was Clostridiales, while the most significant biomarkers at T2, T3, and T4 were Bacilli, Escherichia, and Peptostreptococcaceae, respectively. This suggested that therapeutic injury of the conditioning regimen and antibiotics during the conditioning phase had already made the gut microbiota evolve into a “pathogenic community”, which may promote the onset of aGvHD.

As cytokines reflect the systemic immune response and the functions of different immune cell populations related to aGvHD, exploring both microbiota‐cytokine interaction patterns and aGvHD pathogenesis is crucial. Thus, in addition to the dynamic changes in microbiota, related cytokines have also been explored at different timepoints. At *T1, roseburia* was found to be related to a decreased risk of aGvHD. In vitro, Roseburia has been shown to be capable of producing butyrate.^36^ Butyrate‐producing bacteria have been demonstrated to be associated with lower incidence of GvHD. At this timepoint before conditioning, Doki et al also indicated that a higher tendency of *Bacteroidetes* was a protective factor for the development of acute GvHD.^37^ At T2, we showed that a higher abundance of *Acinetobacter johnsonii* was associated with a higher incidence of acute GvHD. Furthermore, *Acinetobacter johnsonii* was negatively correlated with the secretion of anti‐inflammatory IL‐4 and TNF‐α. In a previous study, CD4^+^CD25^+^FoxP3^+^ regulatory T cells (Tregs) were shown to effectively prevent GvHD. IL‐4 was shown to drive the expansion of Tregs and mediate the suppression of GvHD.^38^ TNF‐α priming induces Treg proliferation in vivo, whereas it limits the ability of CD4 and CD8 conventional T cells (Tcons) to proliferate and induce GvHD.^39^ Of note, our suggested cytokines mechanisms do not completely correlate with the mechanisms described in some published papers of GvHD pathogenesis.^40^, ^41^, ^42^, ^43^ In fact, TNF‐α has been widely regarded as pro‐inflammatory cytokine and elevated in GvHD pathogenesis.^40^, ^41^ It is speculated that the effect of IL4 and TNF‐α at T2 timepoint is through Tregs. However, Tregs were not measured due to undetectable immune cell count at T2 timepoint when myeloablative conditioning had made bone marrow empty, and this was a limitation of the current study. Another possible explanation was that the cytokine changes at T2 timepoint (the infusion of stem cell day) were not directly associated with the pathogenesis of GvHD. When analyzing the differences in the overall levels of cytokines between the GvHD and non‐GvHD groups, we found that TNF‐α at the T3 timepoint was obviously elevated with significance in GvHD group. At T3, *Rothia mucilaginosa* was demonstrated to be linked with a decreased risk of aGvHD. In addition, *Rothia mucilaginosa* was accompanied by decreased secretion of IL‐8. The level of IL‐8 has been widely accepted as a biomarker of a proinflammatory role in aGvHD.^44^, ^45^ Golob et al. also supported that the microbiota around the time of neutrophil recovery post‐HCT is predictive of the subsequent development of acute GvHD, and the presence of *Actinobacteria* and *Firmicutes* was positively correlated with subsequent GvHD, while Lachnospiraceae was negatively correlated.^32^ At the timepoint of T4, higher abundances of *Lactobacillus paracasei* were identified to have positive correction with GvHD, and *Lactobacillus paracasei* was related to the downgrading of IL‐10. Regarding the immune effect, IL‐10 expanded regulatory donor T cells and played a suppressive role in GvHD.^41^ *Acinetobacter johnsonii* was related to the downregulation of IL‐2 and TNF‐α. IL‐2 has been shown to be pleiotropic and at times exert contradictory effects. This is in part because IL‐2 binds to dimeric receptors of two general types: the IL‐2RαIL2Rγ receptor, which is expressed on activated T cells and Tregs, and the IL2RβIL‐2Rγ receptor, which is expressed on immunologically active T, B, and natural killer (NK) cells.^46^

Numerous studies have explored the predictive role of gut microbiota in aGvHD occurrence and related mortality; however, the conclusions are not entirely consistent. Peled et al found no association with GvHD in a very large cohort, but only to GvHD related mortality.^4^ Peled et al. demonstrated associations between the abundance of a group of bacteria and disease progression after allo‐HCT, but not GvHD.^47^ Other trials relating to microbiome and GvHD did find relation. Mancini et al. has reported a low bacterial alpha‐diversity at 10 days post HSCT was the only variable significantly correlating with an increased risk of GvHD within 30 days.^48^ Holler et al. has revealed the major microbiome shifts in the course of allogeneic SCT are more prominent in association with gut GvHD.^31^ In our study, our longitudinal analysis suggested that the microbiota at different timepoints may be related to the incidence of GVHD.

There are several limitations to the current study that should be addressed in future studies. First, the sample size was not large enough, which might inflate or dilute the impact of microbiota in this cohort. Second, the microbiota data were specifically studied until 30 days after HSCT, which may be an immunologically crucial timepoint; however, subsequent timepoints may also be important. Another limitation of the study is the lack of information about the donor's feces content as a healthy control. Finally, the samples were collected at a single center, which might not be fully comparable to other transplant centers, where diets, lifestyles, ethnicity, microbial pathogen exposure, antimicrobial prophylaxis, empiric treatment, and allo‐HSCT protocols differ. To clarify these differences, multicenter studies are highly needed.

## 5 CONCLUSIONS

In this prospective cohort, we conducted longitudinal analysis of gut microbiota and related cytokines in the pathogenesis of aGvHD post allo‐HSCT. Different gut microbiota associated with the development of GvHD at different timepoints have been described, including pathogenic or protective and possibly related cytokines. The abundances of Roseburia at T1, Acinetobacter johnsonii at T2, Rothia mucilaginos at T3, and Lactobacillus paracasei, Acinetobacter johnsonii at T4 were identified to be related with aGvHD. These findings suggested that manipulation of the gut microbiota at different timepoints during allo‐SCT might be a promising avenue for the prophylaxis and treatment of aGvHD.

## AUTHOR CONTRIBUTIONS

**Ling Qi:** Data curation (lead); formal analysis (equal); methodology (equal); writing – original draft (lead). **Jie Peng:** Data curation (equal); methodology (equal). **Xianbao Huang:** Data curation (equal); methodology (equal). **Ting Zhou:** Data curation (equal). **Genmei Tan:** Data curation (equal); formal analysis (equal). **Fei Li:** Data curation (equal); formal analysis (equal); validation (equal); writing – review and editing (lead).

## CONFLICT OF INTEREST STATEMENT

The authors declare that they have no conflicts of interest.

## ETHICAL APPROVAL AND CONSENT TO PARTICIPATE

Informed consent was obtained from the patient for all treatments.

## Supporting information


Data S1:

## Data Availability

The datasets used and/or analyzed during the current study are available from the corresponding author on reasonable requests.
