# Supplementary material for: Longitudinal dynamics of gut microbiota in the pathogenesis of acute graft‐versus‐host disease
Source: Cancer Med. 2023 Dec 6;12(24):21567–78. doi: 10.1002/cam4.6557 (PMC10757094; doi:10.1002/cam4.6557)
Supplement: Supplementary file 1 — Data S1: [file CAM4-12-21567-s001.docx]

**Table 1. The multivariate analysis of baseline factors between the GvHD and non-GvHD groups**

| **Outcome** | **Hazard ratio (95%Confidence interval)** | ***P* value** |
| --- | --- | --- |
| **Acute GvHD**  Malignant disease status at HSCT (CR vs. non-CR)  Donor type (haploidentical vs. matched sibling)  GvHD prophylaxis (with or without ATG ) | 0.880 (0.271-2.857)  2.683 (0.888-8.106)  0.656 (0.201-2.137) | 0.831  0.080  0.484 |

**Abbreviations:** GvHD, graft versus host disease; ATG, anti-thymocyte globulin; CR, complete remission.

**Table 2. The details about GvHD parameters.**

|  | Details | Number |
| --- | --- | --- |
| GVHD Grade | Grade II  Grade III  Grade IV | N=12  N=19  N=3 |
| Involved Organ | One organ  Two organs  Tree organs | N=18  N=14  N=2 |
| Involved Organ | Skin  Gut  Liver | N=26  N=21  N=5 |

**Note:** The evaluation of acute GvHD was based on the Mount Sinai Acute GvHD International Consortium (MAGIC) criteria.

**Table 3 The association between microbiome and survival outcomes.**

|  | | **3-year OS** | **3-year Relapse** | **3-year TRM** |
| --- | --- | --- | --- | --- |
| **Total cohort** | | 69.1±6.2% | 11.5±0.2% | 18.2±0.3% |
| **T1** | **g_Roseburia** | 64.7±9.1% vs. 73.0±9.0%  *P* = 0.368 | 10.3±0.3% vs. 14.3±0.5%  *P* = 0.684 | 20.7±0.6% vs. 10.7±0.4%  *P* = 0.179 |
| **T2** | **f_Actinomycetaceae** | 75.9±7.9% vs. 64.0±9.2%  *P* = 0.566 | 10.3±0.3% vs. 10.3±0.3%  *P* = 0.982 | 13.8±0.4% vs. 24.5±0.7%  *P* = 0.362 |
|  | **g_Actinomyces** | 75.9±7.9% vs. 64.0±9.2%  *P* = 0.566 | 10.3±0.3% vs. 10.3±0.3%  *P* = 0.982 | 13.8±0.4% vs. 24.5±0.7%  *P* = 0.362 |
|  | **s_Acinetobacter_johnsonii** | 75.9±7.9% vs. 61.0±10.6%  *P* = 0.420 | 13.8±0.4% vs. 6.8±0.2%  *P* = 0.377 | 10.3±0.3% vs. 24.1±0.7%  *P* = 0.107 |
|  | **s_Actinomyces_odontolyticus** | 75.9±7.9% vs. 64.0±9.2%  *P* = 0.566 | 10.3±0.3% vs. 10.3±0.3%  *P* = 0.982 | 13.8±0.4% vs. 24.5±0.7%  *P* = 0.362 |
| **T3** | **f_Streptococcaceae** | 74.2±8.5% vs. 68.8±9.3%  *P* = 0.799 | 10.7±0.4% vs. 11.1±0.4%  *P* = 0.945 | 10.7±0.4% vs. 18.5±0.6%  *P* = 0.699 |
|  | **g_Streptococcus** | 74.2±8.5% vs. 68.8±9.3%  *P* = 0.799 | 10.7±0.4% vs. 11.1±0.4%  *P* = 0.945 | 10.7±0.4% vs. 18.5±0.6%  *P* = 0.699 |
|  | **s_Rothia_mucilaginosa** | 61.4±9.9% vs. 81.5±7.5%  *P* = 0.147 | 14.3±0.5% vs. 7.4±0.3%  *P* = 0.428 | 22.4±0.7% vs. 11.1±0.4%  *P* = 0.289 |
|  | **s_Streptococcus_oralis** | 63.6±9.2% vs. 79.1±8.6%  *P* = 0.147 | 10.7±0.4% vs. 11.1±0.4%  *P* = 0.945 | 21.4±0.6% vs. 7.4±0.3%  *P* = 0.085 |
| **T4** | **f_Moraxellaceae** | 68.8±8.2% vs. 68.0±10.4%  *P* = 0.711 | 12.5±0.4% vs. 8.3±0.3%  *P* = 0.578 | 18.8±0.5% vs. 21.1±0.7%  *P* = 0.946 |
|  | **s_Lactobacillus_paracasei** | 69.8±7.0% vs. 67.3±13.6%  *P* = 0.800 | 11.6±0.2% vs. 7.7±0.6%  *P* = 0.657 | 18.6±0.4% vs. 15.4±1.1%  *P* = 0.817 |
|  | **s_Acinetobacter_johnsonii** | 69.2±8.3% vs. 67.9±10.0%  *P* = 0.914 | 14.7±0.4% vs. 4.5±0.2%  *P* = 0.226 | 14.7±0.4% vs. 22.7±1.0%  *P* = 0.308 |

**Note:** In each sheet, the outcome was grouped by lower relative abundance vs. higher relative abundance of microbiome.

**Figure 1 The cumulative incidence of grade II-IV and III-IV aGvHD.**

**Note: The left figure (A) represented grade II-IV aGvHD, and the right figure (B) represented grade III-IV** **aGvHD**.

**Figure 2. The correlation between gut microbiota and immune cell subsets, cytokines and other clinical predictors at four timepoints.**

**Fig.1B**

**Fig.1A**


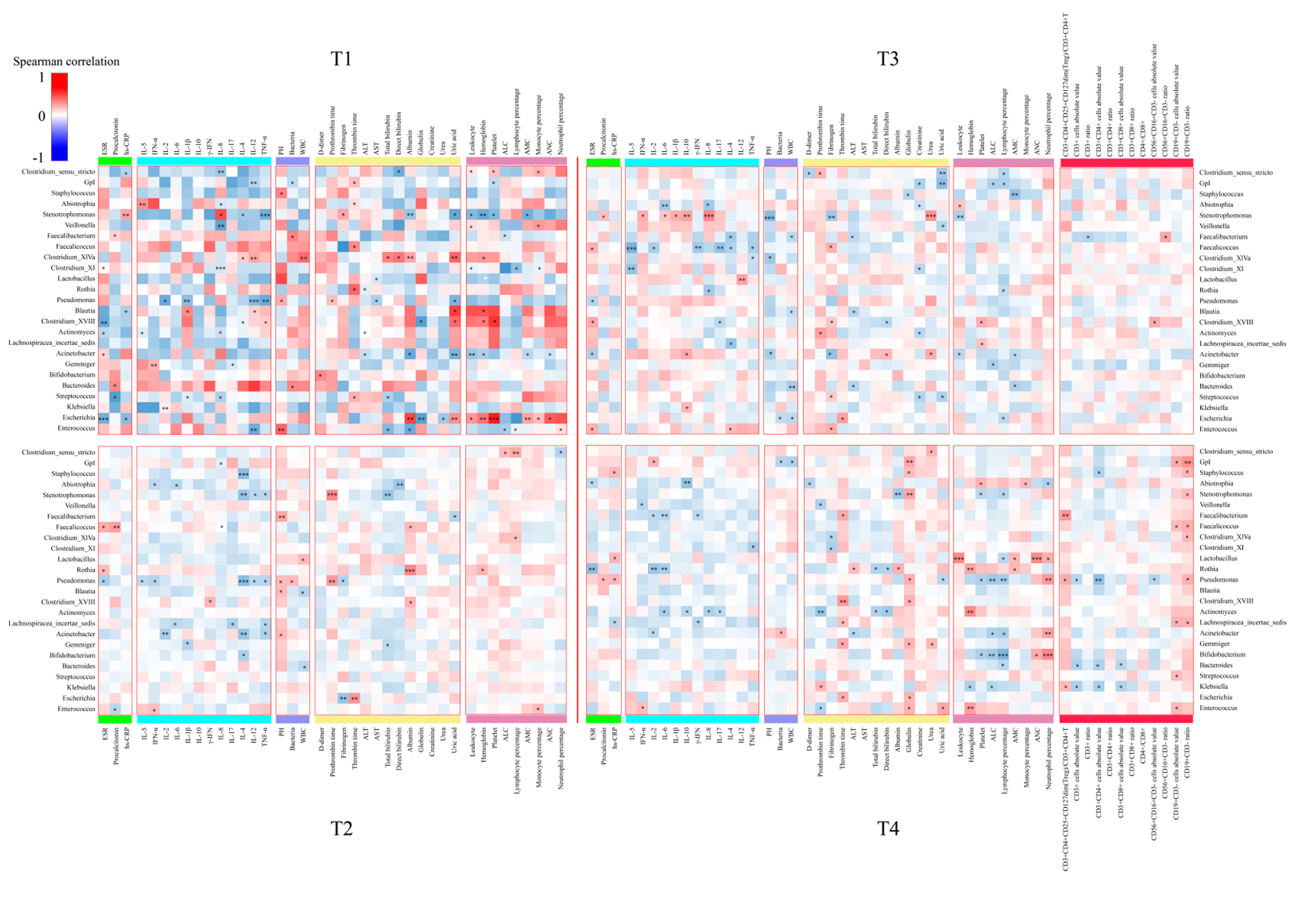


**Note: The figure has shown the correlation between gut microbiota and immune cell subsets, cytokines and other clinical predictors at T1, T2, T3 and T4 timepoints.**
